# Treating Viral Exacerbations of Chronic Obstructive Pulmonary Disease: Insights from a Mouse Model of Cigarette Smoke and H1N1 Influenza Infection

**DOI:** 10.1371/journal.pone.0013251

**Published:** 2010-10-12

**Authors:** Carla M. T. Bauer, Caleb C. J. Zavitz, Fernando M. Botelho, Kristen N. Lambert, Earl G. Brown, Karen L. Mossman, John D. Taylor, Martin R. Stämpfli

**Affiliations:** 1 Medical Sciences Graduate Program, McMaster University, Hamilton, Canada; 2 Department of Pathology and Molecular Medicine, Centre for Gene Therapeutics, McMaster University, Hamilton, Canada; 3 Molecular Biology Undergraduate Program, McMaster University, Hamilton, Canada; 4 Department of Biochemistry, Microbiology and Immunology, University of Ottawa, Ottawa, Canada; 5 Department of Biochemistry and Biomedical Sciences, McMaster University, Hamilton, Canada; 6 AstraZeneca R&D Lund, Lund, Sweden; 7 Department of Medicine, McMaster University, Hamilton, Canada; University of Alabama-Birmingham, United States of America

## Abstract

**Background:**

Chronic obstructive pulmonary disease is a progressive lung disease that is punctuated by periods of exacerbations (worsening of symptoms) that are attributable to viral infections. While rhinoviruses are most commonly isolated viruses during episodes of exacerbation, influenza viruses have the potential to become even more problematic with the increased likelihood of an epidemic.

**Methodology and Principal Findings:**

This study examined the impact of current and potential pharmacological targets namely the systemic corticosteroid dexamethasone and the peroxisome proliferator-activated receptor- gamma agonist pioglitazone on the outcome of infection in smoke-exposed mice. C57BL/6 mice were exposed to room air or cigarette smoke for 4 days and subsequently inoculated with an H1N1 influenza A virus. Interventions were delivered daily during the course of infection. We show that smoke-exposed mice have an exacerbated inflammatory response following infection. While smoke exposure did not compromise viral clearance, precision cut lung slices from smoke-exposed mice showed greater expression of CC (MCP-1, -3), and CXC (KC, MIP-2, GCP-2) chemokines compared to controls when stimulated with a viral mimic or influenza A virus. While dexamethasone treatment partially attenuated the inflammatory response in the broncho-alveolar lavage of smoke-exposed, virally-infected animals, viral-induced neutrophilia was steroid insensitive. In contrast to controls, dexamethasone-treated smoke-exposed influenza-infected mice had a worsened health status. Pioglitazone treatment of virally-infected smoke-exposed mice proved more efficacious than the steroid intervention. Further mechanistic evaluation revealed that a deficiency in CCR2 did not improve the inflammatory outcome in smoke-exposed, virally-infected animals.

**Conclusions and Significance:**

This animal model of cigarette smoke and H1N1 influenza infection demonstrates that smoke-exposed animals are differentially primed to respond to viral insult. While providing a platform to test pharmacological interventions, this model demonstrates that treating viral exacerbations with alternative anti-inflammatory drugs, such as PPAR-gamma agonists should be further explored since they showed greater efficacy than systemic corticosteroids.

## Introduction

Viral infections have been implicated as a major cause of chronic obstructive pulmonary disease (COPD) exacerbations. These episodes lead to declined lung function and concomitant acute deteriorations in respiratory health [1]. Hospitalization rates have been shown to increase during the influenza season, particularly in unimmunized COPD populations [2]. These episodes place an exorbitant burden on health care systems and lead to similarly high patient mortality.

While cigarette smoking is the primary etiological factor for COPD itself [3], exacerbations are caused predominantly by viral and bacterial infections [4], with as many as 40 to 60% of all exacerbations attributed to respiratory viral infections alone [5], [6], [7]. While rhinoviruses and respiratory syncytial virus are responsible for the largest fraction of those exacerbations, seasonal influenza has also been shown to be a significant cause of acute episodes. Unfortunately, while the relative contribution of influenza virus to COPD exacerbations is thought to be less serious in regions that offer immunization [8], and immunization is currently the GOLD standard preventative treatment, some patients believe it may trigger exacerbations and thus refuse vaccination [9], [10], making it a persistent threat.

Few studies have examined the effects of cigarette smoking on antiviral responses *in vivo*. We have previously shown that a high dose influenza infection following smoke-exposure causes increased inflammation without impairing influenza-specific immunologic memory [11], providing further support for the efficacy of vaccination. More recently, Gualano and colleagues showed the inflammatory response that ensues following smoke-exposure does not protect against influenza virus infection, but rather worsens the host response [12]. In a study by Kang *et al.* cigarette smoke was shown to selectively augment the airway- and alveolar- inflammatory and remodeling responses elicited by viral pathogen- associated molecular patterns and influenza A virus [13]. These studies suggest that cigarette smoke-induced inflammation plays a defining role in the initial inflammatory responses to influenza infection.

The objective of this study was to examine how therapeutics could target inflammatory responses in smoke-exposed virally-infected mice. We hypothesized that targeting the excessive inflammation observed in these animals would lead to a better clinical outcome. To test our hypothesis, we exposed mice to cigarette smoke for four days, which we have previously shown causes a mild inflammation [14], and subsequently infected them with a mouse-adapted H1N1 influenza A virus. Dexamethasone, a corticosteroid, and pioglitazone, a peroxisome proliferator-activated receptor (PPAR)-gamma agonist, were delivered daily. We show that pioglitazone had greater efficacy in smoke-exposed influenza-infected mice when compared to the systemic steroid intervention. Collectively, these data demonstrate that while the PPAR-gamma agonist, pioglitazone, significantly attenuates inflammatory responses and was superior to the effects systemic corticosteroids elicited in this model; alternative anti-inflammatory therapies may thus lead to new interventions for the treatment of viral exacerbations of COPD. Some of the results of these studies have been previously reported in the form of abstracts [15], [16], [17].

## Results

### Cigarette smoke exacerbates the inflammatory response to influenza A virus infection

**Figure 1:**
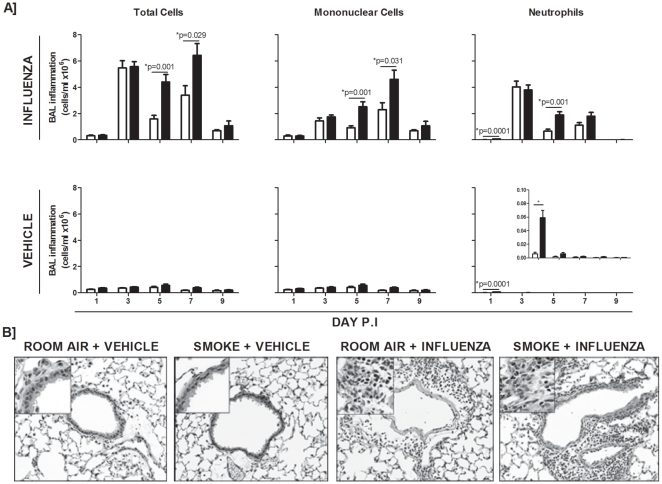
Influenza infection of smoke-exposed animals. C57BL/6 mice were exposed to room air (open bars) or cigarette smoke (closed bars). Subsequently, mice were given a PBS vehicle or inoculated with influenza A virus intranasally. (A) Broncho-alveolar lavage (BAL) samples were obtained and total cells were enumerated at the indicated times post-infection. Mononuclear cell and neutrophil differentials were also assessed. (B) Light photomicrographs of representative hematoxylin and eosin –stained cross sections of lung tissue were taken at 5 days post-infection. Data are presented as means ± SEMs for n = 3–12 animals per group.

Viral infections are well understood to exacerbate inflammation in smokers [18]. While we and others have shown that cigarette smoke exacerbates the inflammatory response to influenza virus infection [11], [12], [13], the whole body smoke-exposure system used in this study has not, to our knowledge, been shown to exacerbate the inflammatory response to influenza infection in mice. Thus, to assess if inflammation was altered in virally-infected smoke-exposed animals, mice were exposed to cigarette smoke and subsequently inoculated with a mouse-adapted H1N1 influenza A virus. Room air-exposed controls and smoke-exposed animals showed no differences in the total number of inflammatory cells recovered in the broncho-alveolar lavage (BAL) at both one and three days after infection with influenza virus (Figure 1A, upper panel). We observed an exaggerated inflammatory response in smoke-exposed influenza-infected animals compared to controls at five days post-infection, comprised of increased numbers of both mononuclear and neutrophilic cells. Representative light photomicrographs of formalin fixed lung tissues from all groups showed this exaggerated inflammation at five days post-infection in smoke-exposed influenza-infected mice (Figure 1B). We also observed greater inflammation in smoke-exposed influenza-infected animals seven days post-infection (Figure 1A). As previously reported, this model of smoke-exposure causes significant neutrophilic inflammation, which subsides following smoking cessation ([14], and Figure 1A, inset graph).

### Clearance of influenza and type I interferon responses are not impaired in smoke-exposed animals

**Figure 2:**
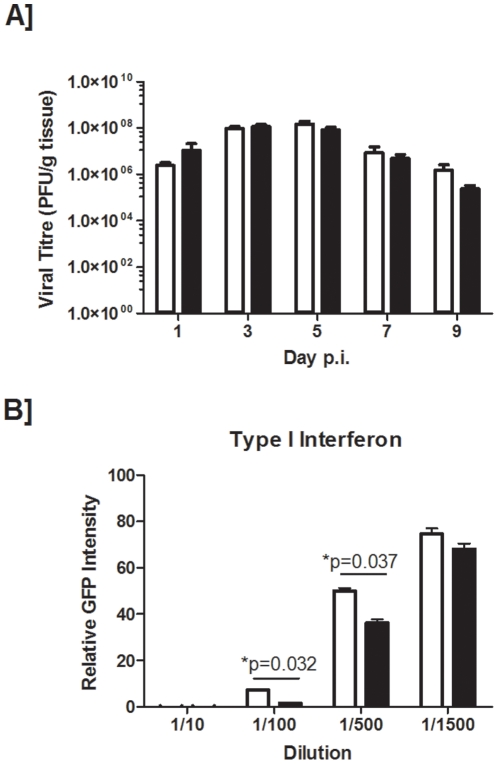
Viral titres and type I interferon responses. C57BL/6 mice were exposed to room air (open bars) or cigarette smoke (closed bars). Mice were infected with influenza A virus. (A) Viral titres were determined in lung homogenates at the indicated times post-infection. (B) The presence of type I interferons at day 3 post-infection was also determined by plating the indicated dilutions of BAL on confluent monolayers of IRF-3 deficient mouse embryonic fibroblasts and performing a vesicular-stomatitis virus (VSV) plaque reduction assay. 100% relative intensity indicates the absence of type I interferon activity, whereas 0% relative intensity indicates sufficient levels of type I interferon to completely prevent VSV-GFP replication. Data are presented as the means ± SEMs for n = 3–17 animals (A), and n = 5 animals (B) per group.

To determine if the exacerbated inflammation observed in smoke-exposed animals was the result of greater virus replication, we assessed viral titres in the lung. We observed no difference in the amount of virus recovered from room air and smoke-exposed animals (Figure 2A). Along similar lines, when we assessed type I interferon activity (IFN is an antiviral cytokine detrimental to the control of viral infection), we observed greater IFN activity in the BAL of influenza-infected smoke-exposed animals than in that of influenza-infected control mice (Figure 2B). Taken together, these data suggest that cigarette smoke exacerbates the inflammatory response to influenza A virus without impairing antiviral immune responses.

### Precision cut lung slices elaborate increased levels of pro-inflammatory chemokines

**Figure 3:**
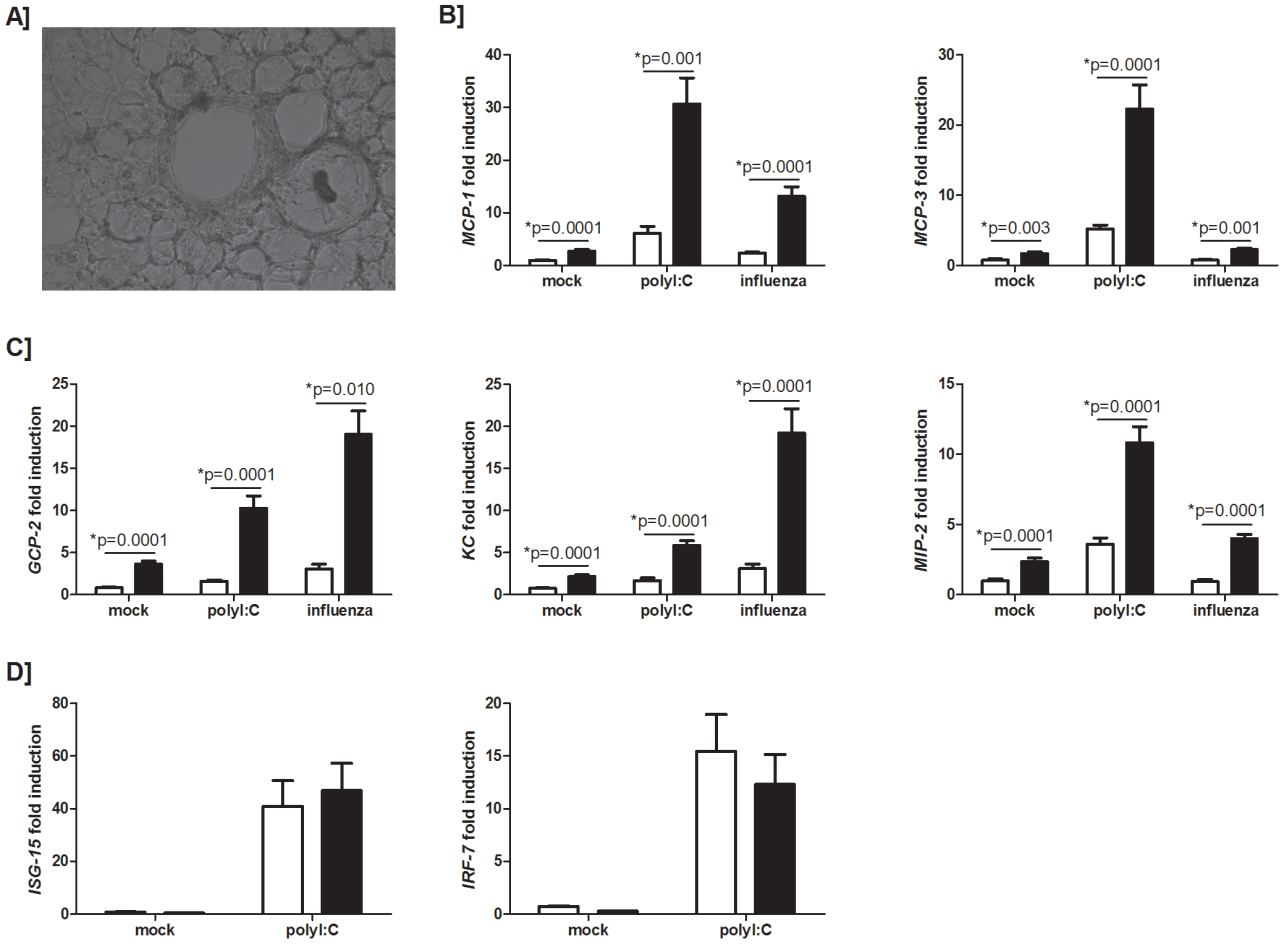
Expression of mediators from precision cut lung slices. C57BL/6 mice were exposed to room air (open bars) or cigarette smoke (closed bars). (A) Precision cut lung slices were generated and cultured *ex vivo*. Induction of MCP-1 and MCP-3 (B) and GCP-2, KC, and MIP-2 (C) transcripts, normalized to levels of a house keeping gene, GAPDH, in the same sample and expressed relative to an untreated sample, were obtained from dsRNA (polyI:C) stimulated and influenza infected precision cut lung slices by real-time quantitative RT-PCR. Induction of ISG-15 and IRF-7 were determined similarly (D). Data are presented as means ± SEMs for n = 5–15 lung slices generated from 3 independent experiments (polyI:C studies) and a representative experiment (influenza studies).

Since an increased viral burden in smoke-exposed animals did not appear to be contributing to the exaggerated inflammation observed in those animals, it is plausible to argue that a smoke-exposed lung is responding differently to a viral stimulus, which may be contributing to the exaggerated response. To assess the immunological state of the lung at the time of viral infection we generated precision cut lung slices (PCLS), as this unique and informative tool would allow us to examine the impact of a viral stimulus on the lung *resident* cells without further considering the impact of cells that have been recruited to the lung from the periphery. PCLS (Figure 3A) from the lungs of room air- and smoke- exposed animals were stimulated *ex vivo* with the dsRNA ligand, polyinosinic polycytidylic acid (polyI:C) and influenza A virus, and expression of key mediators was assessed by real-time quantitative RT-PCR. Basal expression levels of monocyte chemotactic proteins (MCP)-1 and MCP-3 (Figure 3B), and granulocyte chemotactic protein (GCP)-2, KC, and macrophage inflammatory protein (MIP)-2 (Figure 3C) were significantly greater in mock treated smoke-exposed lung slices. We observed a significantly greater induction of MCP-1 and MCP-3, GCP-2, KC, and MIP-2 in response to polyI:C stimulation of smoke-exposed lung slices compared to controls (Figure 3B and 3C, respectively). Smoke-exposed lung slices infected *ex vivo* with influenza virus also had significantly greater induction of MCP-1, MCP-3, GCP-2, KC, and MIP-2 compared to influenza infected room air controls. In contrast to our *in vivo* type I IFN data (refer to Figure 2B), we observed no significant differences in the levels of induction of interferon stimulated gene (ISG)-15 or interferon regulatory factor (IRF)-7 from smoke-exposed PCLS compared to controls when stimulated with polyI:C (Figure 3D). These data demonstrate that a smoke-exposed lung is differentially primed when compared to controls to respond to a viral insult, since increased expression of inflammatory mediators is observed, despite equivocal levels of antiviral type I interferons. Importantly, these findings provided the rationale to assess if targeting the inflammatory response, as opposed to the viral burden, would improve the inflammatory outcome of infection in smoke-exposed animals.

### Administration of corticosteroids does not beneficially impact the outcome of influenza infection in smoke-exposed animals

**Figure 4:**
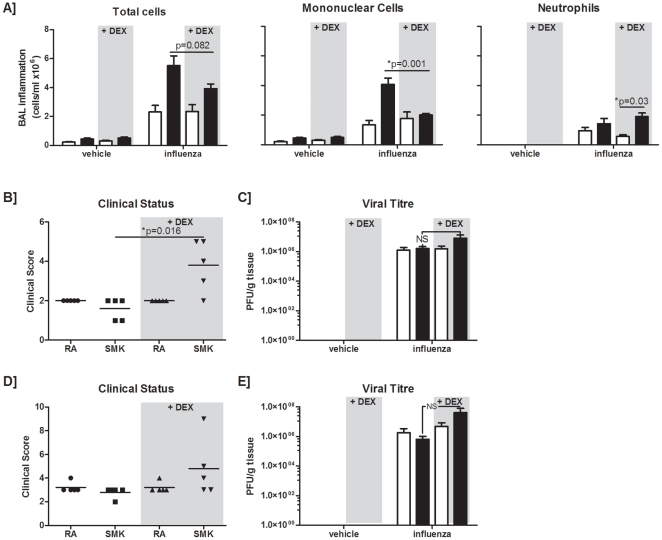
Effect of dexamethasone in smoke-exposed influenza-infected mice. Female C57BL/6 mice were exposed to room air (open bars) or cigarette smoke (closed bars). One hour prior to treatment with vehicle or inoculation with influenza A virus, mice were gavaged with 3mg/kg of dexamethasone (dex). Mice were gavaged daily with 3 mg/kg of dex. (A) BAL samples were obtained at 5 days post-infection and total cells were enumerated. Mononuclear cell and neutrophil differentials were also assessed. Clinical presentation in animals at the time of sacrifice (B), and viral titres (C) were also determined. Clinical presentation (D), and viral titres (E) were also assessed in mice sacrificed at day 7 post-infection. Data are presented as the means ± SEMs for n = 4–6 animals per group.

Since systemic corticosteroids are currently recommended in the treatment of exacerbations of COPD [19], [20] and considering that we observed dysregulated inflammatory mediator expression in smoke-exposed animals, we wanted to assess if systemic delivery of dexamethasone would impact the exacerbated inflammation observed following viral infection of smoke-exposed animals. Mice were given a previously established dose of oral corticosteroids [21] one hour prior to viral infection and daily thereafter. In-line with previously published data [22], [23], dexamethasone was not able to attenuate influenza-induced inflammation five days post-infection (Figure 4A). Although dexamethasone significantly attenuated BAL mononuclear inflammation in smoke-exposed influenza-infected mice, it had no significant impact on overall inflammation in the BAL (p = 0.082) and in fact resulted in worsened clinical presentation (Figure 4A and B). We did observe a trend toward increased viral burden in smoke-exposed dexamethasone-treated animals (Figure 4C), and therefore sought to determine if dexamethasone treatment had a detrimental impact later in the course of infection. Although one dexamethasone-fed smoke-exposed influenza-infected animal reached end-point seven days post-infection, these animals did not have significantly worsened clinical presentation or increased viral titres (Figure 4D and E, respectively). Thus, systemic corticosteroid delivery may not be a favorable treatment in the context of an influenza virus infection of smoke-exposed mice.

### The PPAR-gamma agonist pioglitazone ameliorates the outcome of infection in smoke-exposed animals

**Figure 5:**
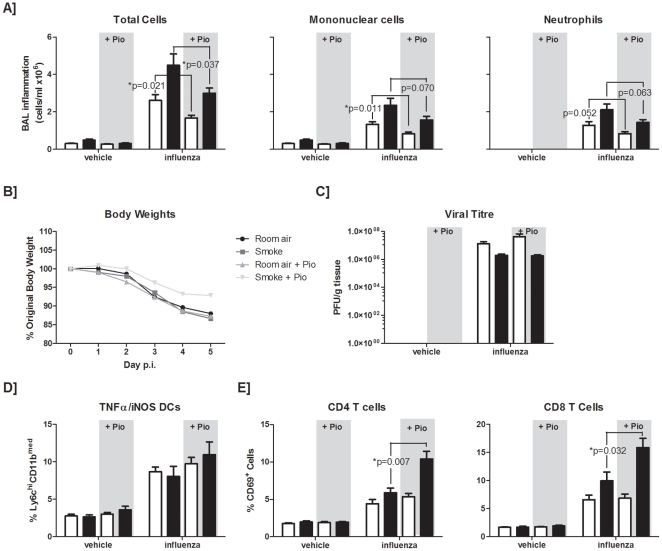
Effect of a PPAR-γ agonist, pioglitazone, on antiviral responses in smoke-exposed mice. C57BL/6 mice were exposed to room air (open bars) or cigarette smoke (closed bars). Starting on the second day of smoke exposure, mice were gavaged daily with 60 mg/kg of pioglitazone (Pio). Following four days of smoke-exposure, mice were given a sterile vehicle or inoculated with influenza A virus intranasally. (A) Broncho-alveolar lavage (BAL) samples were obtained at 5 days post-infection and total cells were enumerated. Mononuclear cell and neutrophil differentials were also assessed. (B) Body weight was monitored throughout the course of the viral infection and expressed relative to body weight on the day 0 of infection. (C) Viral titres were also determined. Percentages of TNFα/iNOS producing dendritic cells (D), and activated CD4+ and CD8+ T cells (E) were obtained from the live gate of CD45+ cells. Data are presented as the means ± SEMs for n = 5–6 animals per group.

Since corticosteroids were shown to be only partially effective in controlling the exacerbated inflammatory response to influenza infection in smoke-exposed mice and considering that the clinical outcome in mice was not entirely favorable, we sought to assess whether an alternative anti-inflammatory strategy shown to be effective during influenza infection would treat the increased inflammation observed in smoke-exposed animals. Recently, Aldridge *et al.* published that the type II diabetes drug, pioglitazone, a PPAR-gamma agonist, was effective in attenuating inflammatory responses in mice infected with influenza [24]. Pioglitazone along with another PPAR-gamma agonist, rosiglitazone, have also had a beneficial effect in animal models of neutrophilic inflammation [25], [26]. We demonstrated with a previously established dose [24] that pioglitazone-fed room air control animals had significantly attenuated (p = 0.021) total numbers of inflammatory cells in the BAL following infection compared to vehicle-fed mice (Figure 5A). While numbers of mononuclear cells were significantly reduced (p = 0.011), neutrophils recovered from the BAL were only modestly attenuated (p = 0.052) in those animals. Given that pioglitazone ameliorates the inflammatory outcome following infection with this mouse-adapted H1N1 influenza virus, we delivered pioglitazone to our smoke-exposed influenza-infected animals and found that the exaggerated inflammation observed in smoke-exposed virally infected animals could be significantly attenuated. In contrast to influenza-infected control animals, smoke-exposed mice did not have significantly attenuated mononuclear (p = 0.07) or neutrophilic (p = 0.063) inflammation. All influenza infected animals lost body weight during the course of infection; however, pioglitazone-treated smoke-exposed mice lost less than did their control-treated counterparts (Figure 5B). In line with observations made by Aldridge *et al.*, pioglitazone did not impact viral clearance in control mice, and furthermore did not alter clearance in our smoke-exposed animals (Figure 5C).

Since pioglitazone treatment altered the excessive inflammation observed in smoke-exposed virally-infected animals, we wanted to further characterize what effect pioglitazone was having on different cell types within the lung. We thus carried out an extensive flow cytometric analysis of whole lung digests. Pioglitazone had been previously reported to attenuate the TNF-α/inducible nitric oxide synthase (iNOS)-producing dendritic cell (tipDC) populations [24], thus we also examined whether this population may play a role in attenuating inflammation in our smoke-exposed animals. We were unable to detect any differences in percentages of this population in pioglitazone-treated controls, or in pioglitazone-treated smoke-exposed animals (Figure 5D). We did not observe any differences in B cells (data not shown); however, we did observe significantly greater percentages of both activated CD4^+^ and CD8^+^ T cells in smoke-exposed pioglitazone-treated animals compared to controls (Figure 5E), despite no changes in the percentages of these cell types (data not shown). Taken together, these data suggest a role for PPAR-gamma agonists as anti-inflammatory agents that are able to impact the excessive inflammation observed in smoke-exposed influenza-infected animals without impairing viral clearance.

### CCR2 deficiency does not have a beneficial effect on influenza-related inflammation in smoke-exposed hosts

**Figure 6:**
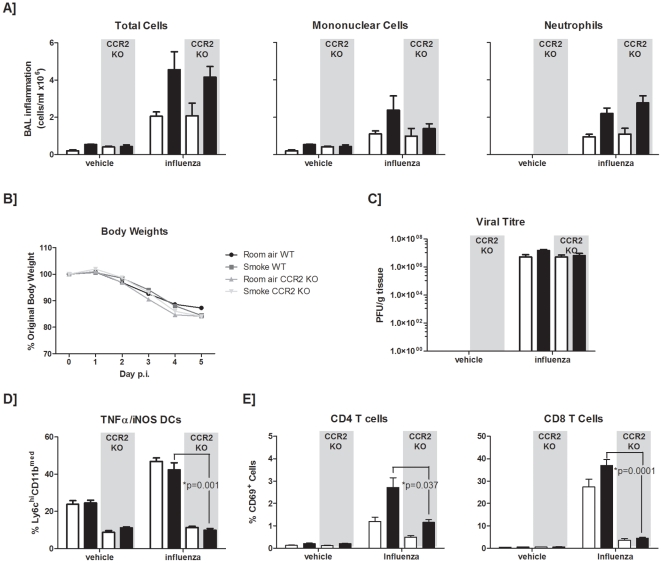
Effect of a CCR2 deficiency on antiviral responses in smoke-exposed mice. C57BL/6 wild-type (WT) or CCR2 knock-out (KO) mice were exposed to room air (open bars) or cigarette smoke (closed bars). Subsequently, mice were given a sterile vehicle or inoculated with influenza A virus intranasally. (A) Broncho-alveolar lavage (BAL) samples were obtained at 5 days post-infection and total cells were enumerated. Mononuclear cell and neutrophil differentials were also assessed. Percent body weight change throughout the viral infection was documented (B), and viral titres were also determined (C). Percentages of TNFα/iNOS producing dendritic cells (D), and activated CD4+ and CD8+ T cells (E) were obtained from the live gate of CD45+ cells. Data are presented as the means ± SEMs for n = 5 animals per group.

Given that pioglitazone has been shown to attenuate levels of cytokines that act through the CCR2 receptor, and several studies have shown that a deficiency in CCR2 signaling ameliorates the detrimental effects of influenza infection [24], [27], [28], we assessed whether blocking CCR2 signaling would lead to a better outcome in our smoke-exposed mice. Interestingly, complete absence of the CCR2 receptor did not attenuate the exaggerated inflammation observed in the BAL of smoke-exposed influenza-infected animals (Figure 6A). While the level of mononuclear cells was attenuated, greater numbers of neutrophils were found in the BAL of CCR2 deficient smoke-exposed, influenza-infected animals than in wild-type controls. In addition, body weight loss and viral titres were unchanged in smoke-exposed influenza-infected CCR2 deficient mice compared to controls (Figure 6B and C). In a similar fashion to our PPAR-gamma agonist experiments, we assessed whether there were any observable differences in cell types within the lung of the CCR2-deficient animals by a flow cytometric analysis. While a CCR2 deficiency led to attenuated percentages of tipDCs in influenza-infected mice compared to controls (Figure 6D), as previously reported [24], percentages of CD69^+^, CD4 and CD8 T cells were significantly attenuated in CCR2 deficient mice (Figure 6E). Thus, while the PPAR-gamma agonist, pioglitazone, significantly attenuated exaggerated inflammatory responses in smoke-exposed mice, a CCR2 deficiency did not appear to confer any beneficial effect on smoke-exposed influenza-infected animals.

## Discussion

It is well understood that viral infections trigger COPD exacerbations [29]. Given the increased likelihood of an influenza epidemic, and the contribution of influenza viruses to COPD exacerbations, the objective of this study was to test current and novel therapeutics for their ability to reduce the exaggerated inflammation observed in virally-infected smoke-exposed animals. Ultimately understanding how we may alter anti-influenza responses in the lung of smoke-exposed mice will hopefully translate to promising pharmacological intervention strategies that may be used in the treatment of viral-induced exacerbations of COPD.

Using a mouse model of whole-body cigarette smoke exposure to test our hypothesis, we show that animals mount an exaggerated inflammatory response to a mouse-adapted influenza A virus. We have previously demonstrated that four days of smoke exposure in this system causes a mild neutrophilic inflammation within the lung of C57BL/6 mice [14]. In addition, the protocol of smoke exposure has been previously validated and shown to achieve blood carboxyhaemoglobin levels that are comparable to those found in regular human smokers [21]. While it might be argued that four days of smoke exposure is a minimal exposure protocol, we also observed exaggerated inflammatory responses to influenza A virus with eight weeks of smoke exposure (unpublished observation). Given that COPD is a very complex disease, modeling the aspects that include, but are not limited to, emphysema, bronchitis and bronchiolitis, which contribute to disease pathogenesis in humans, has to our knowledge never been achieved in concert in mice. Kang *et al.* recently demonstrated that early innate immune events were detrimental to the development of emphysema in smoke-exposed mice [13], providing further rationale for examining the impact of acute cigarette smoke exposure to H1N1 influenza antiviral responses.

Few studies in the literature have examined the effects of cigarette smoke exposure on antiviral responses. Recently, Gualano and colleagues showed that smoke-exposed BALB/c mice that were infected with influenza A virus had greater viral burden at early timepoints (day 3) post-infection [12]. In contrast, we did not observe increased titres in our smoke-exposed animals. While their study argued that increased titres may have been the result of decreased type I interferon responses, we observed greater type I interferon activity in our smoke-exposed mice. Furthermore, our precision cut lung slice data also suggested that type I interferon responses are in-tact in smoke-exposed lungs as we did not observe any differences in the induction of key interferon stimulated genes, ISG-15 and IRF-7, in response to stimulation with dsRNA ligands. Because these studies used different smoke-exposure systems, strains of mice and viruses, direct comparison of these data are difficult; however, it should be noted that we have previously reported greater levels of type I interferons using a nose-only smoke exposure system with the parental strain of our virus [11]. In agreement with our data, a study by Kang and colleagues also reported similar viral titres in smoke-exposed animals compared to controls at all time-points post-infection [13]. Taken together, our data suggest that innate type I interferon-mediated antiviral responses are not impaired in smoke-exposed mice. Furthermore, it is unlikely that the initial viral burden in smoke-exposed animals is leading to the excessive inflammation observed at later times post-infection.

Having established in this study that cigarette smoke was impacting the inflammatory response to influenza A virus, and not initial replication and clearance of the virus, and since mounting evidence suggests that COPD exacerbations are inflammatory events (reviewed in [29], [30], [31]), we chose to focus the remainder of this study on addressing whether exaggerated inflammation could be attenuated. Using PCLS, we were able to ascertain that the resident cells within the smoke-exposed lung were producing greater transcript levels of inflammatory chemokines in response to a dsRNA stimulus and to an influenza virus infection *ex vivo*. These data provided further evidence to support the notion that the smoke-exposed lung is differentially primed to respond to an inflammatory stimuli, given that basal levels of all transcripts measured were significantly greater in smoke-exposed compared to room air control PCLS.

There are a number of treatments that reduce the incidence and severity of COPD exacerbation, included among these are inhaled and oral steroids, combinations of inhaled steroids and beta agonists [32], as well as the anti-muscarinic Tiotropium [33]. Thus, we first sought to assess if first-line treatments used in the clinic, such as systemic corticosteroids, could ameliorate inflammatory outcomes in influenza-infected smoke-exposed mice. Although it is understood that treating the cytokine storm observed during an influenza infection with steroids is in-effective [22], [34], no studies to our knowledge involving models of cigarette smoke and virus have addressed if a steroid intervention would control the exacerbated responses observed in smokers. To this end, we delivered a dose of corticosteroids, which we had previously shown in a model of cigarette smoke and non-typeable *Haemophilus influenzae* infection to attenuate exaggerated inflammatory responses [21]. While the steroid intervention failed to attenuate inflammation, we did observe a significantly worsened clinical presentation in mice early in the course of influenza infection (day five). Although at day seven we did not observe a statistically worsened clinical presentation in our smoke-exposed influenza-infected mice, one mouse of five did reach endpoint. Along similar lines, the viral titres observed at both days five and seven post-infection did not reach statistical significance; in contrast, in our model of cigarette smoke exposure and *Haemophilus influenzae* infection, systemic corticosteroid treatment significantly impaired bacterial clearance from the lungs of infected mice. Given that current corticosteroid therapies are well understood to reduce the frequency of COPD exacerbation [32], it is plausible that in light of our contrasting data in a viral versus bacterial model that systemic corticosteroids are preferentially effective in decreasing the inflammation associated with a given type of exacerbating insult, a hypothesis that would require further investigation.

Since we showed that steroid interventions did not play a role in attenuating the inflammation observed in this model of cigarette smoke exposure and influenza infection, we examined whether another anti-inflammatory agent would attenuate the observed dysregulation in the inflammatory response. PPAR-activators are putative drug targets in COPD [35], [36], [37], yet no models of cigarette smoke exposure and viral or bacterial infection have examined their potential. In addition, PPAR-gamma agonists have been shown to exert beneficial effects in models of acute respiratory distress syndrome [38], lipopolysaccharide-induced lung neutrophilia [25], and lethal influenza A virus challenge [24]. Given the compelling evidence that these agonists could provide important relief of inflammation during times of viral exacerbations, we chose to administer a dose of the PPAR-gamma agonist, pioglitazone, to our mice that had been previously shown to ameliorate the outcome of a lethal influenza virus infection [24]. Importantly, we showed that this agonist is able to significantly attenuate exaggerated inflammatory responses in the BAL of smoke-exposed influenza-infected mice. Despite the fact that pioglitazone significantly attenuated the total number of cells lavaged in the BAL, only a moderate attenuation of the mononuclear cells (p = 0.073) and neutrophils (p = 0.063) was observed. While it remains arguable that the method of delivery and/or dosing of the drug may have contributed to the efficacy of the response, these possibilities are being further explored, as the beneficial effects of PPAR-gamma agonists have been ascertained in other studies that utilized intravenous delivery of lower drug doses (0.3 mg/kg) [38], and oral delivery of different formulations (rosiglitazone) [25].

While Aldridge and colleagues argue that PPAR-gamma agonists exert their effects during an influenza infection by down-regulating the recruitment of TNF-α/i-NOS producing dendritic cells from the bone marrow [24], we did not observe any changes in this cell population. This discrepancy may be explained by the fact that we did not inoculate our mice with a lethal dose of influenza. Interestingly, following an extensive flow cytometric analysis of lung digests, the only significant differences observed were on the percentage of activated (CD69^+^) CD4 and CD8 T cells. As previously reported in influenza-infected room air and smoke-exposed mice [11], and amongst influenza-infected pioglitazone-treated animals (data not shown), percentages of both subsets of T cells remained unchanged. The increased percentage of activated T cells, to our knowledge, has never been reported following pioglitazone administration in smoke-exposed influenza-infected animals. While pioglitazone was able to ameliorate the weight loss observed in influenza-infected animals this observation was not entirely surprising as PPAR-gamma agonists are known to cause weight gain [39]. When considering anti-inflammatory therapies it is imperative to ensure that host defense mechanisms are not impaired; in that regard, pioglitazone did not alter clearance of the virus from our smoke-exposed animals. The fact that effector CD4 and CD8 T cells are required for the clearance of RNA virus infections, the increased percentages of activated CD4 and CD8 T cells likely plays a imperative role in clearing influenza viruses from the lung despite altering the inflammatory cells recruited to the lung.

Since pioglitazone is known to exert some of its effects through the CCR2 receptor, we next sought to examine if a deficiency in this receptor would attenuate exaggerated inflammation in our smoke-exposed virally-infected animals. We showed that smoke-exposed CCR2-deficient mice did not have attenuated inflammation in response to influenza infection. To assess if there were any observable differences between our influenza-infected smoke-exposed pioglitazone-treated and CCR2-deficient animals that could account for the differences observed in total BAL inflammation, we carried out a comprehensive flow cytometric analysis of whole lungs, and found more activated CD4 and CD8 T cells within the lungs of our smoke-exposed and influenza-infected animals treated with pioglitazone. While CD8^+^ T cells have been shown to be critical for clearance of respiratory viruses, depending on the number of CD8^+^ T cells and overall immune status, these cells may cause severe lung pathology. While CD8^+^ T cells have been implicated in mice and in humans to contribute to tissue damage, further studies would be required to assess if this increase in activated T cells has any implications to changes in a smoke-exposed lung.

Taken together, the results of our study demonstrate a striking difference between the inflammatory responses mounted by smoke- and room air-exposed mice to viral challenge. Data obtained from PCLS further highlights the notion of a skewed inflammatory profile in the lung of smokers. In addition, this study brings to light the potential for PPAR-gamma agonists as anti-inflammatory agents for the treatment of inflammation associated with exacerbations of COPD, and demonstrates the value of mouse models of cigarette smoke exposure and viral infection for pre-clinical screening of potential therapeutics for patients with stable COPD and for those suffering from exacerbations of their disease.

## Materials and Methods

### Animals

6–8 week old female C57BL/6 mice were purchased from Charles River Laboratories (Montreal, PQ, Canada) and kept on a 12-h light-dark cycle with unlimited access to food and water. Cages, food, and bedding were autoclaved. All studies were approved by the McMaster University Animal Research Ethics Board. Body weight and clinical presentation of animals were monitored daily in each study, as previously described [40].

### Cigarette Smoke Exposure

Mice were exposed to mainstream tobacco smoke using a whole body smoke exposure system (SIU48, PROMECH LAB AB, Vintrie, Sweden), as previously reported [21]. Briefly, mice were exposed to 12 2R4F reference cigarettes with filters removed (Tobacco and Health Research Institute, University of Kentucky, Lexington, KY, USA) twice daily, 5 days per week, for approximately 50 minutes. An acclimatization period of three days prepared mice for smoke exposure and has been previously described [41]. Mice were exposed to 4 days of cigarette smoke and were no longer exposed following influenza infection. Control animals were exposed to room air only.

### Influenza Infection

Isoflurane-anesthetized mice were intranasally infected with 50 PFU of a mouse-adapted H1N1 influenza A (A/FM/1/47-MA) virus in 35 µl of phosphate-buffered saline (PBS) vehicle. Control animals received 35 µl of PBS vehicle. A/FM/1/47-MA is a fully sequenced, plaque-purified preparation that is biologically characterized with respect to mouse lung infections [42]. Animals were not exposed to cigarette smoke on the day of viral delivery or for the entire course of the viral infection.

### Pioglitazone and Dexamethasone Treatment

Mice were treated with a previously established dose [24] of 60 mg/kg pioglitazone (Toronto Research Chemicals, Toronto, Canada), suspended in 100 µL of PBS, via oral gavage beginning on day 2 of smoke-exposure and daily thereafter. 3 mg/kg of dexamethasone, a dose previously established in smoke-exposed animals [21] was suspended in 200 µL of PBS and delivered via oral gavage to mice one hour prior to viral inoculation and daily thereafter. Control mice were gavaged with 100 µL or 200 µL of phosphate buffered saline (PBS), respectively, for the pioglitazone and dexamethasone studies.

### Collection and Measurement of Specimens

Lungs were removed, tracheas cannulated (Becton Dickinson and Co., Sparks, MD, USA), and a lobe from the right lung was removed for determination of viral titre. Broncho-alveolar lavage (BAL) was generated by lavaging the lung twice with PBS (250 µl followed by 200 µl). Total cell counts from the BAL were determined by haemocytometer. BAL cells were pelleted by centrifugation and differential cell counts were obtained from cytospins that were generated after re-suspension of cell pellets. Cytospin cells were stained by Hema 3 (Biochemical Sciences INC., Swedesboro, NJ, USA). Differential counts were determined by counting at least 300 cells. Standard hemocytological criteria were used to classify mononuclear cells and neutrophils. The remainder of the right lung was used for flow cytometric analysis, and the left lung lobe was inflated with formalin for histological assessment.

#### Measurement of Viral Titre

Lung homogenates were used to determine viral titre on Madin-Darby Canine Kidney (MDCK) monolayers. MDCK cells were maintained in α-MEM supplemented with 10% fetal bovine serum, 1% L-glutamine, and 1% penicillin/streptomycin. MDCK cells were seeded into 6 well dishes. Confluent monolayers of MDCK cells were washed twice with PBS to remove serum. Serial dilutions of lung homogenate samples were prepared and 200 µl of each was added to the MDCK monolayers. Dishes were rocked and incubated at 37°C for 30 minutes. Agarose overlays (1∶1 ratio of 1.3% agarose (Biotechnology grade; Bioshop Canada INC., Burlington, Canada), and 2× MEM/F-11 supplemented with 2% L-glutamine and 2% penicillin/streptomycin, containing 1 µg/ml trypsin-EDTA) were applied and agarose allowed to solidify before replacing dishes to 37°C. Two days later, plaque assays were fixed using Carnoy's Fixative (75% methanol and 25% glacial acetic acid). Plaques were enumerated and viral titres were expressed as the number of infectious plaque forming units per gram of lung tissue.

### Histological Assessment

The right lung lobe was inflated with 10% formalin at a constant pressure of 20 cm of H_2_O. The inflated lung was fixed for an additional 72 hours in 10% formalin. Tissues were transferred into 70% ethanol and prepared for paraffin embedding. 3 µm cross-sections were cut and stained with hematoxylin and eosin. Images were captured using a Leica camera and microscope (Leica Microsystems, Richmond Hill, Canada). OpenLab software (version 3.0.3, Improvision, Guelph, Canada) was used for photo acquisition.

### Flow Cytometric Analysis

Lungs were cut into ∼2 mm pieces, shaken for 1 h at 37°C in 150 U/ml collagenase III (Gibco, Burlington, Canada) in HBSS, pressed through nylon mesh, washed twice in HBSS, and counted on a hemacytometer. 10^6^ cells were plated in 96-well, round-bottom plates. Cells were washed, and re-suspended in 25 µl of staining cocktail for 30 min at 4°C. Antibodies used in this study include anti-CD11c (FITC, HL3, BD Biosciences, San Jose, CA, USA), anti-CD4 (PE-610, RM4-5, Invitrogen, Burlington, Canada), anti-CD69 (PerCP-Cy5.5, H1.2F3, BD Biosciences San Jose, CA, USA), anti-CD45R (APC-Alexa Fluor 750, RA3-6B2, eBiosciences, San Diego, CA, USA), anti-CD3 (Pacific Blue, eBio500A2, eBiosciences, San Diego, CA, USA), and anti-CD8 (Pacific Orange, 5H10, Invitrogen, Burlington, Canada), as well as appropriate isotype control antibodies (BD, San Jose, CA, USA and eBiosciences, San Diego, CA, USA). For analysis of TNF-α/iNOS producing dendritic cells, 10^6^ cells were incubated for 4 ½ hours in 50 ng/ml PMA and 500 ng/ml ionomycin. Cells were subsequently surface stained with anti-Ly6c (AlexaFluor 647, BD Biosciences, San Jose, CA, USA) and CD11b (PE, BD Biosciences, San Jose, CA, USA), followed by fixation and permeabilization with BD cytofix/cytoperm (BD Biosciences, San Jose, CA, USA), and intracellular staining for iNOS (FITC, BD Biosciences, San Jose, CA, USA) and TNF-α (PE-Cy7, BD Biosciences, San Jose, CA, USA). Fluorescence minus one staining cocktails and isotype control antibodies (BD Biosciences and eBiosciences) were used to distinguish background staining from positive signals. Flow cytometric data was acquired using an LSRII flow cytometer (BD Biosciences, San Jose, CA, USA). Data were analyzed with FlowJo Software (Tree Star, Ashland, OR, USA).

### Lung Slicing and Culture

Lungs were sliced using a modification to a standard protocol that has previously been described [43]. The protocol was adapted within our laboratory and others [44]. After sacrifice, the tracheas were cannulated using an intravenous catheter (20 G Intima, Becton, Dickinson, Sandy, UT, USA), and the chest walls were removed. Lungs were inflated with approximately 1.4 ml of agarose (type VII-A low gelling temperature; Sigma Aldrich, St. Louis, MO, USA) that was warmed to 37°C and prepared to a concentration of 2% in Hank's buffered saline solution (HBSS), supplemented with N-2-hydroxyethlypiperazine-N'-2-ethanesulphonic acid (HEPES) (0.2 M, pH 7.4). Subsequently, 0.2 ml of air was injected into the lung in order to flush the agarose-HBSS solution out of the conducting airways. The agarose was allowed to gel by cooling the lung to 4°C for 15 minutes. The lung lobes were dissected away and a flat surface was cut on the lobe parallel and caudal to the main bronchus. The lung lobes were maintained in an ice-cold 1× HBSS solution prior to and during slicing. 120 µm thick slices were generated using a vibratome (Leica; model VT 1000S, Richmond Hill, Canada) at 4°C. Approximately 40 slices were isolated from each mouse lung.

Lung slices were subsequently transferred to and cultured in Dulbecco's Modified Eagles Medium (DMEM)/F12 (Gibco, Burlington, Canada) supplemented with 35 µg/ml L-Ascorbic Acid (Sigma-Aldrich, Oakville, Canada), 5 µg/ml Transferin (Gibco, Burlington, Canada), 2.85 µg/ml Insulin (Sigma-Aldrich, Oakville, Canada), and 3.25 ng/ml Selenium (atomic absorption standard solution; Sigma-Aldrich, Oakville, Canada). The solution was filter-sterilized using a 0.22 µm pore filter. The DMEM/F12 solution was further supplemented with 250 ng/ml Amphotericin B (Sigma-Aldrich, Oakville, Canada) and 1% Penicillin/streptomycin. The medium was changed every 1 h for the first 3 h of culture in order to remove any remaining agarose and cell debris from the lung slice culture. Lung slices were stimulated the next day for 6 hours with 100 ug/ml of dsRNA mimetic polyinosinic-polycytidylic acid (GE Healthcare, Mississauga, Canada) that was reconstituted in phosphate buffered saline, for 24 hours with 200 PFU/ml of a mouse-adapted H1N1 influenza A (A/FM/1/47-MA) virus, or were left untreated. Samples were collected in RNA later (Ambion, Austin, TX, USA) and preserved at -80°C until extraction of RNA.

### RNA extraction and Real-Time Quantitative RT PCR

Lung slices were collected and placed into 200 µl of RNAlater (Qiagen, Mississauga, ON, Canada), and stored at −80°C until further use. RNA was extracted from the lung slices according to the animal tissues protocol from the RNEasy Kit (Qiagen, Mississauga, ON, Canada). Optional on-column DNase digestion was performed. RNA was quantified using the Agilent 2100 Bio-analyzer (Agilent Technologies, Mississauga, ON, Canada). The quantity and integrity of isolated RNA was determined using the Agilent 2100 Bioanalyzer (Agilent, Palo Alto, CA, USA). Subsequently, 100 ng of total RNA was reverse-transcribed using 100 U of Superscript II (Invitrogen, Burlington, Canada) in a total volume of 20 µL. Random hexamer primers were used to synthesize cDNA at 42°C for 50 min, followed by 15 min incubations at 70°C. Real-time quantitative RT-PCR was performed in triplicate, in a total volume of 25 µl, using a Universal PCR Master Mix (Applied Biosystems, Foster City, CA, USA). Primers for ISG-15, IRF-7, MCP-1, MCP-3, GAPDH, along with FAM-labeled probes were purchased from Applied Biosystems. PCR was performed using the ABI PRISM 7900HT Sequence Detection System using the Sequence Detector Software version 2.2 (Applied Biosystems, Foster City, CA, USA). Data were analyzed using the delta, delta Ct method. Briefly, gene expression was normalized to the housekeeping gene (GAPDH) and expressed as fold change over the control group (room air control, mock).

### VSV-GFP plaque reduction assays

Type I interferon bioactivity was assessed in BAL samples by plaque reduction assay as previously described [11]. Briefly, interferon regulatory factor 3-deficient mouse embryonic fibroblasts were seeded into 24 well dishes. Serial dilutions of BAL fluid were prepared and incubated with the cells for 18 hours. Cells were subsequently washed with 37°C PBS and incubated with vesicular stomatitis virus expressing GFP (VSV-GFP; kindly provided by Dr. Brian Lichty, McMaster University) for 40 minutes in serum-free media. The viral inoculum was removed and replaced with DMEM containing 1% methylcellulose. GFP fluorescence intensity was measured 24 hours later on a Typhoon Trio imager (GE Healthcare, Mississauga, Canada) and quantified using the ImageQuant™ TL software (GE Healthcare, Mississauga, Canada).

### Data analysis

Data are expressed as mean ± SEM. Statistical analysis was performed with SPSS statistical software, version 17.0 (Chicago, IL, USA). We assessed significance (p<0.05) using the SPSS Univariate General Linear Model, t-tests were subsequently performed for two-group comparisons.
